# SERS-Enabled Direct Detection of Furfural in a Complex Oil Mixture

**DOI:** 10.3390/s26185705

**Published:** 2026-09-08

**Authors:** Xiaoqin Zhang, Hongbin Zhu, Hao Liu, Jin Cao, Han Shi, Shanyuan Niu

**Affiliations:** 1 Electric Power Research Institute of State Grid Jiangsu Electric Power Co., Ltd., Nanjing 211103, China; 15850736276@126.com (X.Z.); njzhb@msn.cn (H.Z.); 2 College of Engineering and Applied Sciences, Nanjing University, Nanjing 210026, China; haoliu@smail.nju.edu.cn (H.L.); 502025340017@smail.nju.edu.cn (J.C.); 602025340064@smail.nju.edu.cn (H.S.)

**Keywords:** surface-enhanced Raman spectroscopy, furfural, direct oil-sample analysis, transformer insulation aging, vibrational spectroscopy

## Abstract

Furfural is a pivotal indicator of the aging condition of transformer oil-paper insulation. Traditional analytical techniques such as liquid chromatography require sophisticated pretreatment and phase-separation procedures and are therefore not well suited to rapid oil-sample analysis. This study reports the direct analysis of furfural in complex oil mixtures using surface-enhanced Raman spectroscopy (SERS). The weak vibrational response of furfural in oil was enhanced using a Au-coated silicon nanowire substrate fabricated by metal-assisted chemical etching (MACE). The textured metal-coated surface enabled trace furfural at the μL/L level to be measured directly in the oil mixture without adsorption enrichment or chemical extraction, with the entire test process completed within 1 min. Peak deconvolution was used to extract the fitted area of the 1365 cm^−1^ band, and the relationship between this Raman response and furfural concentration yielded R^2^ = 0.9910. Temperature- and pressure-dependent measurements were also performed to examine their effects on the positions of the main Raman peaks. This work demonstrates a rapid approach for analyzing trace furfural in complex liquid mixtures and provides a basis for further spectroscopic studies of transformer oil-paper insulation aging.

## 1. Introduction

As power systems expand and renewable energy integration increases, transformers have become pivotal components of the modern power grid [1]. Their operational stability is inextricably linked to the safety and reliability of the entire electrical system [2]. In China and worldwide, a large installed fleet of oil-immersed transformers has entered long-term service, making insulation aging assessment increasingly important [3]. Insulation degradation is the primary precursor to transformer failure; if left undiagnosed, it can lead to catastrophic insulation breakdown and equipment decommissioning [4]. Consequently, evaluating the state of transformer oil-paper insulation—specifically by monitoring chemical aging byproducts—is of critical engineering significance [5]. Among these indicators, furfural, a characteristic byproduct of cellulose thermal decomposition, is a pivotal biomarker whose concentration directly reflects the degradation level of the insulation system [6].

Current detection methodologies for furfural in oil primarily rely on Liquid Chromatography (LC), Infrared Spectroscopy (IR), and Photoacoustic Spectroscopy (PAS) [7]. While LC offers high precision [8], it is inherently constrained by an inefficient “separation-before-detection” workflow, involving complex multi-step pretreatment that results in detection cycles measured in hours [9]. Conversely, while IR and PAS techniques offer faster response times [10], they face severe challenges in complex oil matrices: IR suffers from intense C-H stretching backgrounds that mask characteristic signals, and both methods struggle with multi-component deconvolution due to broad, overlapping spectral envelopes and low signal coupling efficiency. These limitations make laboratory-grade offline tools difficult to adapt to rapid analysis of transformer oil samples [11].

In contrast, Raman spectroscopy provides a high-resolution, non-destructive “intrinsic fingerprint” for molecular identification, offering the distinct advantage of simultaneous multi-component co-detection without the need for oil-gas separation [12,13,14]. However, the inherently small Raman scattering cross-section of target molecules limits detection sensitivity in complex oil environments, making the identification of trace-level aging products challenging [15]. To overcome this bottleneck, Surface-Enhanced Raman Spectroscopy (SERS) has emerged as a transformative solution, utilizing Localized Surface Plasmon Resonance (LSPR) on metallic nanostructures to amplify vibrational signals by several orders of magnitude [16].

In recent years, several research groups have explored the application of Surface-Enhanced Raman Spectroscopy (SERS) for detecting aging by-products like furfural in transformer oil. For instance, Chen Weigen’s team achieved Raman identification of furfural via adsorption on silver nanosheets [17], while other researchers have improved sensitivity through extraction-combined SERS strategies [18]. Although these studies laid a vital foundation for the spectral identification of aging products, they typically rely on prolonged molecular adsorption/enrichment times or complex extraction processes to obtain stable signals. Such workflows, characterized by low detection efficiency and cumbersome sample preparation, struggle to meet the urgent demands for real-time, on-site monitoring in modern power grids.

To address the dual challenges of detection feasibility [19] and quantitative accuracy [20], this study uses a Au-coated silicon nanowire SERS substrate fabricated by metal-assisted chemical etching (MACE). The MACE-generated textured silicon architecture and Au coating provide the SERS-active surface for direct Raman measurements in the oil phase. Beyond advances in SERS substrates and sample-preparation strategies, recent studies have also explored noise management using two-dimensional materials [21] and machine-learning-assisted label-free SERS for reliable prediction of dissolved furfural in transformer oil [22].

In this work, furfural at the μL/L level was analyzed directly in oil samples without adsorption enrichment, chemical extraction, or complex surface modification. Compared with previously reported approaches that require a 5 min substrate–oil contact period or specific molecular functionalization such as 4-ATP-mediated capture, the present method directly analyzes untreated oil using a high-enhancement Au-coated MACE silicon nanowire substrate, with the entire workflow completed within 1 min. We also investigated the effects of temperature (−100 to 90 °C) and pressure on the positions of the main Raman peaks of furfural. In addition, deconvolution-based spectral analysis was used to examine the relationship between the fitted 1365 cm^−1^ Raman response and furfural concentration. These findings provide a basis for rapid analysis of aging products in transformer oil.

## 2. Materials and Methods

### 2.1. Reagents and Instrumentation

Furfural (analytical grade; Shanghai Aladdin Biochemical Technology Co., Ltd., Shanghai, China) was used as the target analyte. A gas-to-liquids (GTL)-based commercial insulating oil, Shell Diala S4 ZX-I (Shell plc, London, UK), served as the transformer-oil matrix. The oil was supplied by the Electric Power Research Institute of State Grid Jiangsu Electric Power Co., Ltd. (Nanjing, China). The typical physical properties reported by the manufacturer include a density of 805 kg m^−3^ at 20 °C, a kinematic viscosity of 9.8 mm^2^ s^−1^ at 40 °C, a flash point of 191 °C, and a pour point of −45 °C. Before sample preparation, the oil was maintained in a sealed container and used without additional treatment.

Two Raman spectroscopy systems were employed: one for the fundamental study of pure furfural, and the other for the enhanced detection of trace furfural in oil. The first system was a confocal Raman spectrometer (Horiba, Ltd., Kyoto, Japan) equipped with 532 nm and 785 nm excitation lasers and a temperature-controlled stage (range: −195 °C to 500 °C, accuracy: 0.5 °C). This system was used to analyze the intrinsic Raman characteristic peaks of pure furfural and the influence of temperature on signal intensity and peak position. The system featured a confocal microscopic optical path with a spatial resolution better than 2 μm and a spectral resolution of approximately 4 cm^−1^.

The second system was a portable fiber-optic Raman spectrometer (Ocean Optics QE Pro, Orlando, FL, USA; 638 nm excitation). The laser power was set to 30 mW with an integration time of 30 s and a spectral resolution better than 8 cm^−1^. This system was utilized for enhanced Raman signal testing of trace furfural in oil to evaluate the performance of the Raman enhancement substrate in complex oil matrices.

Pure furfural was characterized using 785 nm excitation to reduce fluorescence interference and obtain a higher-quality reference Raman spectrum. For the portable Raman system intended for potential field measurements, 638 nm excitation was selected by balancing spectral coverage, fluorescence background, Raman signal intensity, and system portability. Therefore, the subsequent furfural-in-oil measurements were performed using the 638 nm portable Raman system.

### 2.2. Fabrication and Characterization of the SERS Substrate

The SERS substrate was fabricated through a patterned silicon route coupled with metal-assisted chemical etching (MACE). First, photoresist was coated on the back side of a cleaned silicon wafer to protect that surface during the subsequent etching steps. On the front side, photolithography was used to define the patterned regions, and inductively coupled plasma (ICP) etching was used to form micrometer-scale grooves. The unetched silicon on the two sides of each groove formed a pair of ridge-like boundaries. Silver nanoparticles were then deposited on the exposed silicon within the grooves as the MACE catalyst. Chemical etching generated dense vertically oriented silicon nanowires in the channels between adjacent ridges. After the MACE step, the photoresist and residual silver nanoparticles were removed. A 50 nm Au layer was subsequently deposited onto the structured silicon surface by electron-beam evaporation at a deposition rate of 0.5 Å s^−1^ to obtain the SERS-active substrate. The morphology of the MACE-formed silicon scaffold was examined by scanning electron microscopy (SEM). The optical response of the SERS substrate was further characterized by UV–Vis total-reflectance spectroscopy over the wavelength range of 200–800 nm. Because the silicon substrate is opaque in this spectral range, the transmittance was approximated as zero, and the absorptance was calculated as A (%) = 100 − R (%).

### 2.3. Acquisition of Pure Furfural Raman Spectra

To obtain the intrinsic Raman signatures of furfural for peak calibration, pure furfural samples were first analyzed. Approximately 5 μL of furfural liquid was dropped onto a clean silicon wafer and placed under the objective lens of the confocal Raman spectrometer, with the laser focused on the droplet surface. The excitation wavelength was set at 785 nm, laser power at 10 mW, and integration time at 10 s. Three accumulations were performed to improve the signal-to-noise ratio (SNR), with a scanning range of 800–1800 cm^−1^.

### 2.4. Detection of Trace Furfural in Oil

An Au-coated silicon nanowire SERS substrate fabricated by metal-assisted chemical etching (MACE) was used to enhance the Raman response of furfural in the oil phase. To evaluate substrate-assisted detection performance, standard furfural oil samples were prepared on a volume/volume (*v*/*v*) basis by directly adding liquid furfural to transformer oil, with concentrations of 10, 50, 100, 200, 400, and 500 μL/L (approximately 11, 55, 110, 220, 440, and 550 mg/L, respectively, based on a furfural density of 1.10 g/cm^3^). Prior to testing, the substrates were cleaned sequentially with acetone, isopropanol, and ultrapure water, then dried under a nitrogen stream. Approximately 5 μL of the furfural-containing oil sample was dropped onto the substrate surface and tested directly using the portable fiber-optic Raman spectrometer probe (Ocean Optics, Orlando, FL, USA). During the experiment, the working distance between the probe and the sample surface was maintained at approximately 6 mm, with a laser power of 30 mW, an integration time of 20 s, and a scanning range of 600–1800 cm^−1^. Blank oil samples were used as controls in each experiment to account for interference from the oil background. The entire test process was completed within 1 min without sedimentation, stirring, or enrichment steps. For each furfural concentration, three measurements were performed using the same substrate. After each measurement, the substrate was cleaned and the oil sample was reloaded before the subsequent measurement.

### 2.5. Data Processing and Quantitative Analysis

The collected Raman spectra were initially subjected to baseline correction using polynomial fitting, followed by smoothing to reduce random noise. For quantitative analysis, the spectral region from 1240 to 1400 cm^−1^ was selected because it contains the main furfural response at 1365 cm^−1^ together with several adjacent oil-matrix-related bands. To resolve the overlapping spectral contributions, peak deconvolution was performed on four bands centered at approximately 1271, 1305, 1337, and 1365 cm^−1^. The fitted peak area of the 1365 cm^−1^ band was used to represent the Raman response of furfural. The relationship between this response and furfural concentration was examined by linear regression, and the coefficient of determination (R^2^) was reported to describe the observed relationship.

### 2.6. Effects of Temperature and Pressure on Raman Peak Positions

Temperature- and pressure-variable experiments were conducted on pure furfural to examine their effects on Raman peak positions.

For temperature-dependent measurements, samples were sealed in quartz capillaries and positioned on a temperature-controlled stage. The temperature was varied from −100 °C to 90 °C, while other parameters remained consistent with Section 2.3.

For pressure-dependent investigations, a diamond anvil cell (DAC) was employed to create a high-pressure environment. The furfural sample was loaded into a micro-hole (typically 100–200 μm in diameter) drilled in a metal gasket, with ruby spheres included as internal pressure calibrants. Raman spectra were collected in-situ as the pressure was incrementally increased.

## 3. Results and Discussion

### 3.1. Spectroscopic Characterization

**Figure 1:**
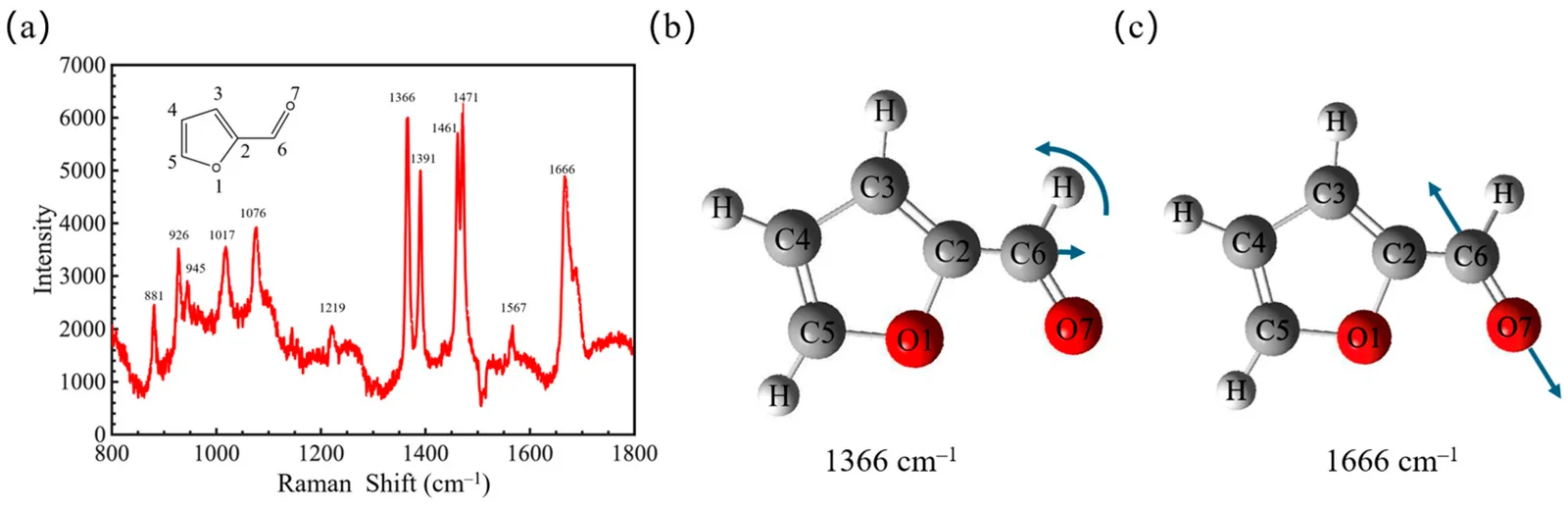
(**a**) Raman spectrum of pure furfural under 785 nm excitation, with the main characteristic peaks labeled. The inset shows the molecular structure and atom numbering of furfural; (**b**) calculated vibrational mode corresponding to the 1366 cm^−1^ band; (**c**) calculated vibrational mode corresponding to the 1666 cm^−1^ band.

The standard Raman spectrum of furfural is illustrated in Figure 1a. Under 785 nm excitation, it exhibits distinct molecular vibrational features within the 800–1800 cm^−1^ range, providing a reliable benchmark for subsequent oil sample detection and quantitative analysis. The primary Raman-active peaks of furfural are concentrated in the mid-to-high wavenumber region, corresponding to the characteristic vibrations of its conjugated carbonyl structure and furan ring skeleton.

In the high-wavenumber region, the peak at 1666 cm^−1^ stands out as the most prominent feature, assigned to the C6=O7 carbonyl stretching vibration. This vibration is accompanied by in-plane scissoring of C6–H and C6–C2, serving as the most signature identification signal for furfural. Due to the high polarizability of the carbonyl π-bond electron cloud, changes in bond length induce dramatic variations in molecular polarizability, resulting in a significantly large Raman scattering cross-section and high spectral intensity. Furthermore, this peak position is highly sensitive to changes in the molecular environment (e.g., conjugation effects, solvent polarity, or metallic adsorption), and is thus regarded as a vital probe for reflecting local electron density distribution and intermolecular interactions.

The adjacent peak at 1567 cm^−1^ is primarily attributed to in-plane ring breathing coupled with C2–C3, C4–C5, and C3–H/C4–H antisymmetric scissoring. This mode encapsulates the collective vibrational characteristics of the aromatic skeleton within the furan conjugated system, representing a typical “breathing-style” expansion and contraction of the ring plane. Its Raman activity originates from the periodic modulation of the skeletal electron density. The peaks at 1461 cm^−1^ and 1471 cm^−1^ form a doublet of comparable intensity, corresponding to ring breathing coupled with C2–C3, C4–C5, and C5–H/C6–H antisymmetric scissoring. These are considered core skeletal fingerprint signals of the furan ring system, reflecting periodic bond length variations and the alternating in-plane swinging of hydrogen atoms. The existence of this doublet arises from coupled modes with slight differences in symmetry; the 1461 cm^−1^ peak is dominated by C–H scissoring, while the 1471 cm^−1^ peak reflects more of the skeletal stretching component. The high Raman intensity in this region suggests significant synergetic changes in polarizability between the ring skeleton and C-H vibrations.

The 1391 cm^−1^ peak also belongs to the ring-breathing category but leans more toward C4–H/C5–H and C2–H/C6–H antisymmetric scissoring. Although the skeletal deformation in this mode is less pronounced, the relative in-plane displacement of hydrogen atoms is more evident, resulting in high intensity and a narrow full-width at half-maximum (FWHM). This peak indicates that while the furan ring maintains an overall conjugated plane, local C–H vibrations undergo energy coupling with the aromatic skeletal stretching, forming a unique vibrational mode.

The characteristic peak at 1366 cm^−1^ exhibits stable intensity and a narrow FWHM, primarily originating from the C6–H in-plane bending vibration. This mode involves the periodic deflection of the aldehydic hydrogen within the molecular plane, accompanied by a slight synergetic displacement of the C6 atom. Since the C=O bond length variation contributes limitedly to polarizability here, the enhancement of this mode stems mainly from local polarizability changes induced by the bending of the C–H bond direction. This peak is viewed as one of the most representative skeletal signals of furfural, providing a key reference for distinguishing different aldehyde-substituted structures.

The peaks at 1219 cm^−1^ and 1076 cm^−1^ are associated with ring breathing coupled with antisymmetric scissoring of C3–H/C5–H and C4–H/C5–H, respectively, serving as auxiliary signals for skeletal identification. In the lower wavenumber region, the 1017 cm^−1^ peak arises from the in-plane symmetric scissoring of C3–H and C4–H, reflecting the synchronous swinging of ring hydrogens. The 945 cm^−1^ peak describes the C6–H out-of-plane wagging; this mode involves the periodic movement of the aldehydic hydrogen away from the molecular plane and is often subject to slight red-shifts due to the polar environment or intermolecular hydrogen bonding. Finally, the 926 cm^−1^ and 881 cm^−1^ peaks are both assigned to in-plane ring breathing, representing low-frequency skeletal modes where ring atoms vibrate synchronously in the radial direction, reflecting the overall response of the ring’s flexible deformation and electronic conjugation.

Overall, the measured standard spectrum of furfural is highly consistent with literature reports, with deviations in major peak positions within a few cm^−1^, confirming the stability and reliability of the testing system. The peaks at 1666 cm^−1^ (C=O stretching) and 1366 cm^−1^ (C–H in-plane bending) are designated as the primary characteristic signals for subsequent oil sample detection.

Vibrational mode diagrams were generated using Gaussian 16 (Gaussian, Inc., Wallingford, CT, USA). As shown in Figure 1b, the 1366 cm^−1^ mode is characterized by the periodic swinging of the aldehydic hydrogen (attached to C6) along a black curve, with the C6 atom slightly shifting outward from the ring. Figure 1c illustrates the 1666 cm^−1^ mode, where the C6 and O7 atoms undergo stretching along the C=O bond axis (blue line). This comprehensive analysis provides a clear spectroscopic basis for identifying furfural and examining its concentration-dependent Raman response, see Table 1.

### 3.2. Structural Characterization and SERS Performance of the Substrate

**Figure 2:**
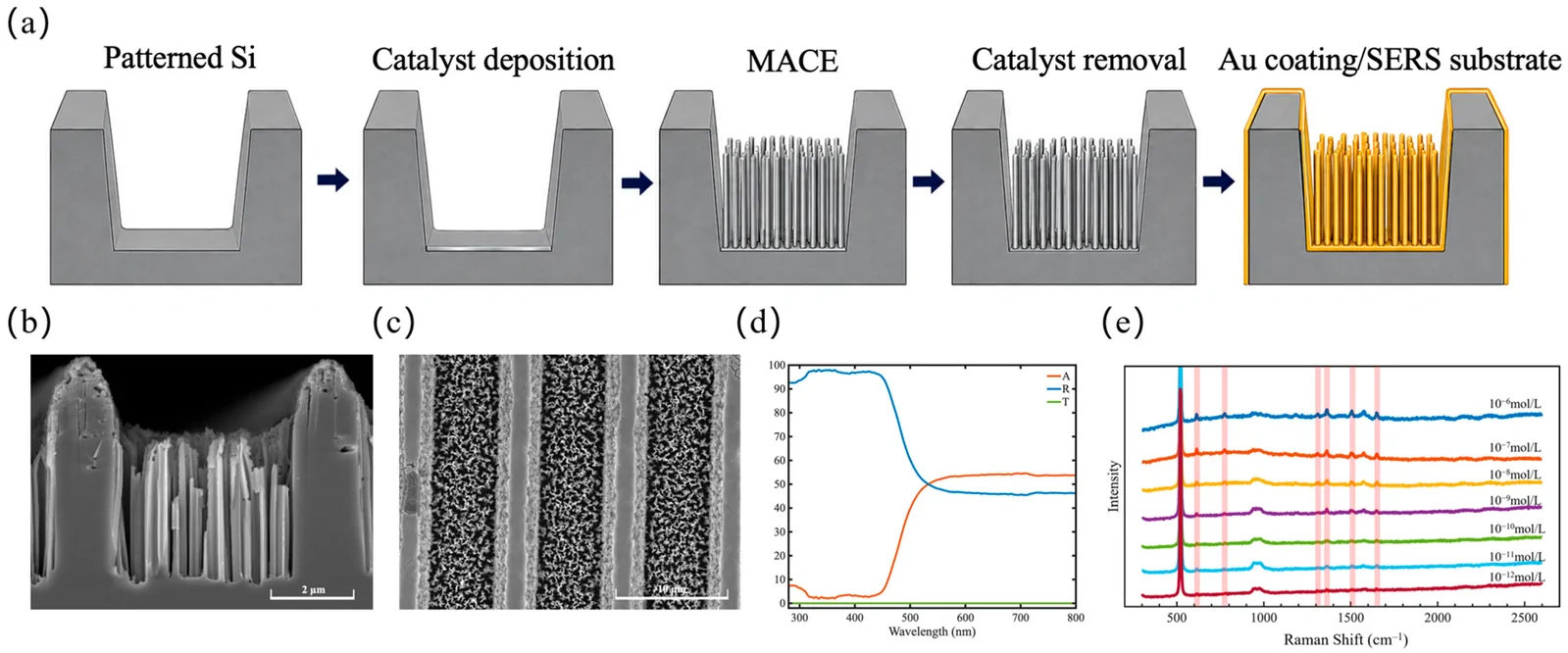
(**a**) Fabrication workflow for the Au-coated MACE silicon nanowire SERS substrate: back-side photoresist protection, front-side photolithographic patterning and ICP formation of grooves, Ag nanoparticle catalyst deposition, MACE formation of silicon nanowires in the grooves, catalyst/photoresist removal, and Au coating; (**b**) cross-sectional SEM image of the MACE-formed silicon scaffold, in which a channel between two ridge-like boundaries contains dense vertically oriented silicon nanowires (scale bar: 2 μm); (**c**) top-view SEM image showing the periodic parallel ridge–channel architecture and the dense nanoscale texture within the channels (scale bar: 10 μm); (**d**) UV-Vis optical response of the prepared SERS substrate, including absorptance, reflectance, and transmittance; (**e**) SERS spectra of R6G solutions measured using the prepared substrate.

The complete fabrication route for the Au-coated MACE silicon nanowire SERS substrate is shown in Figure 2a. A photoresist layer was first applied to the back side of the silicon wafer as an etching-protection layer. Photolithography on the front side defined the patterned regions, and ICP etching produced micrometer-scale grooves. The remaining silicon on both sides of each groove formed ridge-like boundaries. Ag nanoparticles were deposited on the exposed silicon in the grooves and served as the local catalyst during MACE. The repeated ridge–channel units form a periodic microstructured architecture, while each channel contains a dense array of vertically oriented silicon nanowires. Following removal of the photoresist and residual Ag catalyst, Au was coated onto the structured surface to obtain the SERS-active substrate.

The cross-sectional SEM image in Figure 2b shows the MACE-formed silicon scaffold: two ridge-like sidewalls enclose a channel filled with densely packed, vertically oriented nanowires. The matched top-view SEM image in Figure 2c shows the corresponding repeated parallel ridge–channel units and the dense nanoscale texture within the channels. These paired SEM images verify the local ridge–nanowire–ridge morphology generated by the patterned MACE process; they do not, by themselves, establish the thickness or conformity of the final Au coating, or a specific electromagnetic enhancement mechanism. Figure 2d presents the UV–Vis optical response of the Au-coated MACE silicon nanowire substrate. The substrate exhibits appreciable absorptance around 638 nm, indicating effective optical interaction with the excitation wavelength used in the portable Raman system. Figure 2e presents the SERS spectra of Rhodamine 6G (R6G) measured on the prepared substrate as a probe-molecule validation of the Raman response. The enhancement factor (EF) was estimated from the R6G band near 1362 cm^−1^ by comparing 10^−12^ M R6G on the SERS substrate with 10^−7^ M R6G on bare Si. After normalization by the corresponding acquisition times, the estimated EF was approximately 1.1 × 10^5^.

The Raman enhancement of the Au-coated silicon nanowire substrate may arise from the combined contribution of the microstructured surface and nanoscale plasmonic effects. The periodic ridge–channel structure could increase light scattering and optical interaction with the Au-coated surface, while the Au-coated nanowires and the small gaps between adjacent nanostructures may favor localized electromagnetic-field concentration. These structural features are therefore expected to contribute to the enhanced Raman response observed experimentally. Together, the fabrication scheme, the paired SEM images, UV-Vis optical response and the R6G spectra define the structural and functional context for the subsequent furfural measurements. The MACE-derived periodic ridge–channel architecture with nanowires inside each channel provides the structured silicon scaffold for the Au-coated SERS substrate used for direct analysis of trace furfural in complex transformer-oil matrices.

### 3.3. Detection of Furfural in Transformer Oil

Using the Au-coated MACE-fabricated silicon nanowire SERS substrate, the oil sample was deposited onto the active surface and analyzed directly. Identifiable Raman signals were acquired without sample pretreatment, and the entire test process was completed within 1 min, demonstrating rapid analysis of furfural-containing oil samples.

**Figure 3:**
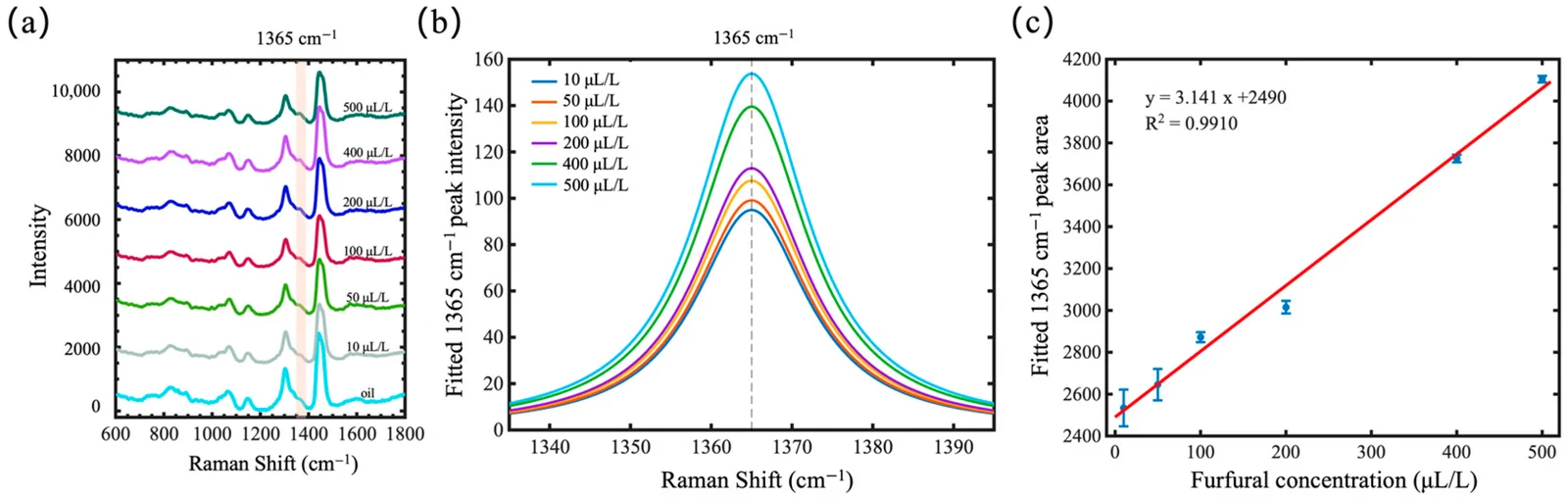
(**a**) SERS spectra of oil samples containing furfural at concentrations of 10, 50, 100, 200, 400, and 500 μL/L, together with the spectrum of the blank oil sample; (**b**) fitted profiles of the 1365 cm^−1^ band obtained from samples with different furfural concentrations; (**c**) relationship between the fitted area of the 1365 cm^−1^ band and furfural concentration. The red line represents the linear regression, y = 3.141x + 2490, with R^2^ = 0.9910.

Figure 3 presents the SERS response of furfural in transformer oil. As shown in Figure 3a, the synthetic oil matrix exhibits several background Raman bands, mainly located at approximately 1071, 1148, 1306, and 1445 cm^−1^, which are attributed to the C-C and C-H stretching and bending vibrations of the oil medium [23]. After adding furfural, a distinct characteristic band appears near 1365 cm^−1^. This band corresponds to the in-plane C-H bending vibration of furfural and can be used as a marker for its identification in oil. As the furfural concentration increases from 10 to 500 μL/L, the response around 1365 cm^−1^ gradually increases, showing that the Au-coated MACE-fabricated silicon nanowire substrate enhances the furfural signal in the complex oil matrix. A discernible response was observed at 10 μL/L without sample pretreatment.

Because direct extraction of the 1365 cm^−1^ peak may be affected by adjacent oil-matrix signals and spectral overlap, the 1240–1400 cm^−1^ spectral window was selected for peak deconvolution. The overlapping bands centered at approximately 1271, 1305, 1337, and 1365 cm^−1^ were fitted, and the fitted area of the 1365 cm^−1^ band was used to represent the furfural Raman response. As shown in Figure 3b, the fitted response generally increases with furfural concentration, indicating a concentration-dependent Raman response after separating the furfural contribution from the overlapping oil-matrix bands.

The relationship between furfural concentration and the fitted 1365 cm^−1^ peak area is shown in Figure 3c. Over the tested concentration range of 10–500 μL/L, the fitted Raman response showed an approximately linear relationship with furfural concentration, described by y = 3.141x + 2490 with R^2^ = 0.9910. This result indicates a concentration-dependent Raman response under the present experimental conditions, rather than establishing a fully validated quantitative calibration model. The relatively large intercept mainly arises from the strong Raman background of the transformer-oil matrix and residual spectral contributions within the fitting window. The reproducibility of the SERS measurements was further evaluated using a 100 μL/L furfural-in-oil sample. The same substrate was measured three times, giving an RSD of approximately 4.1%, while three independently prepared substrate batches showed an inter-batch RSD of approximately 12%. These results describe the observed relationship between the deconvoluted Raman response and furfural concentration in the tested oil samples. Overall, the MACE-SERS method enables rapid, pretreatment-free analysis of trace furfural in transformer oil.

### 3.4. Temperature- and Pressur-Dependent Raman Spectral Changes

We examined the Raman spectra of furfural under variable temperature and pressure conditions, focusing on changes in the positions and profiles of its characteristic peaks. Within the practically relevant range of 20–90 °C, the characteristic Raman peaks showed only minor shifts, supporting the spectral stability of furfural detection. Extreme low-temperature and high-pressure measurements were additionally performed to examine the effects of phase transition and molecular compression on the Raman fingerprint, rather than to simulate normal transformer operation.

**Figure 4:**
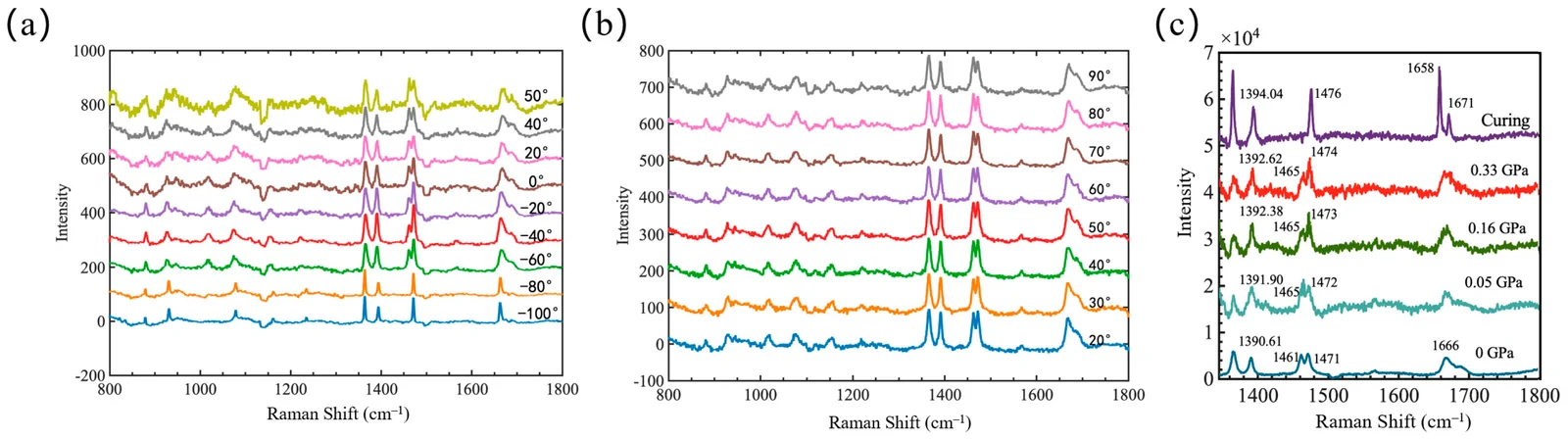
(**a**) Temperature-dependent Raman spectra of furfural in the range of −100 °C to 50 °C; (**b**) Raman spectra of furfural within the typical transformer operating temperature range (20–90 °C); (**c**) pressure-dependent Raman spectra of furfural in the range of 0–0.33 GPa and in the solidified state.

Figure 4a illustrates the temperature-dependent Raman spectra of pure furfural from −100 °C to 50 °C. Between 50 °C and −60 °C, the peak positions remain largely unchanged. When the temperature decreases to approximately −80 °C and below, the furfural sample undergoes a liquid-to-solid phase transition, accompanied by spectral narrowing and changes in peak profiles. A red-shift of approximately 2–4 cm^−1^ is observed for several peaks, while bands near 1461 and 1688 cm^−1^ become less distinguishable. These observations show how low temperature and the associated phase transition affect the positions and profiles of the Raman peaks.

Figure 4b presents the Raman spectra of furfural from 20 °C to 90 °C. Across this temperature range, shifts in the main characteristic peaks are less than 3 cm^−1^, while the peak profiles and relative intensities show limited variation. These results describe the effect of temperature on the observed Raman peak positions under the tested conditions.

Figure 4c presents the pressure-dependent Raman spectra of furfural from 0 to 0.33 GPa, together with the spectrum obtained in the solidified state. Several characteristic bands in the 1300–1700 cm^−1^ region shift slightly toward higher wavenumbers with increasing pressure. The band near 1461 cm^−1^ becomes less distinguishable after further compression and solidification, and the peak near 1666 cm^−1^ splits into components around 1658 and 1671 cm^−1^ under the higher-pressure/solidified conditions. These observations show that pressure affects the positions and profiles of several characteristic Raman peaks of furfural.

## 4. Conclusions

This study developed a Surface-Enhanced Raman Spectroscopy (SERS) system based on a Au-coated silicon nanowire substrate fabricated by metal-assisted chemical etching (MACE) for the rapid analysis of trace furfural in transformer oil. The main findings are summarized as follows:

First, the fabricated Au-coated MACE silicon nanowire substrate combined patterned ridge/sidewall features with vertically textured nanowire regions. The paired cross-sectional and top-view SEM images document the local hierarchical morphology used as the SERS-active surface.

Second, furfural at the lowest experimentally tested concentration of 10 μL/L was directly analyzed in oil samples without extraction or chemical functionalization, and the entire test process was completed within 1 min. Peak deconvolution within the 1240–1400 cm^−1^ spectral window resolved the overlapping bands at 1271, 1305, 1337, and 1365 cm^−1^. The fitted area of the 1365 cm^−1^ band was then used to examine its relationship with furfural concentration. Over the tested range of 10–500 μL/L, linear regression gave R^2^ = 0.9910.

Finally, temperature- and pressure-dependent measurements showed changes in the positions and profiles of the characteristic Raman peaks of furfural. From 20 to 90 °C, shifts in the main peaks were less than 3 cm^−1^. At lower temperatures and under compression/solidification, additional peak shifts and splitting were observed. These results describe the effects of temperature and pressure on the Raman peak positions under the tested conditions.

Future research may further optimize MACE-related structural parameters, including the patterned geometry, etching duration, and Au-coating conditions, to improve morphology uniformity and Raman response reproducibility. Integrating multidimensional spectral variables with advanced machine-learning algorithms may facilitate a more robust quantitative prediction model for furfural. Coupling this SERS detection system with microfluidic modules or fiber-optic sampling structures may further support miniaturized, automated online monitoring of transformer-oil aging products.

Future work may optimize the MACE process and Au-coating conditions to improve structural uniformity and measurement repeatability [24]. Multidimensional spectral analysis and machine-learning algorithms [25] may also be explored to further characterize the relationship between Raman responses and furfural concentration. In addition, coupling the SERS system with microfluidic modules or fiber-optic sampling structures [26] may support the development of miniaturized and automated oil-sample analysis devices.

## Figures and Tables

**Table 1:** Vibrational Mode Assignments of Raman Characteristic Peaks for Furfural.

Peak Position/cm^−1^	Vibrational Mode Assignment
881	In-plane ring breathing of the furan ring
926	In-plane ring breathing of the furan ring
945	C6–H out-of-plane wagging
1017	In-plane symmetric scissoring of C3–H and C4–H
1076	In-plane ring breathing with C4–H/C5–H in-plane antisymmetric scissoring
1219	In-plane ring breathing with C3–H/C5–H antisymmetric scissoring
1366	C6–H in-plane bending
1391	In-plane ring breathing with C4–H/C5–H and C2–H/C6–H antisymmetric scissoring
1461, 1471	In-plane ring breathing with C2–C3/C4–C5 and C5–H/C6–H antisymmetric scissoring
1567	In-plane ring breathing with C2–C3/C4–C5 and C3–H/C4–H antisymmetric scissoring
1666	C6=O7 stretching vibration

## Data Availability

The data presented in this study are available from the corresponding author upon reasonable request.

## Abbreviations

The following abbreviations are used in this manuscript:

SERS	Surface-enhanced Raman spectroscopy
LSPR	Localized surface plasmon resonance
MACE	Metal-assisted chemical etching
R6G	Rhodamine 6G
SEM	Scanning electron microscopy
DAC	Diamond anvil cell

## References

[1] Asl M.B., Fofana I., Meghnefi F. (2024). Review of Various Sensor Technologies in Monitoring the Condition of Power Transformers. Energies.

[2] Jin L., Kim D., Abu-Siada A., Kumar S. (2022). Oil-Immersed Power Transformer Condition Monitoring Methodologies: A Review. Energies.

[3] Liao C., Yang J., Hu X., Qiu Z., Liu X., Zhu W. (2024). Transformer Fault Diagnosis Method Based on Characteristic Quantity Screening of Dissolved Gases in Oil. Electr. Power Eng. Technol..

[4] Lundgaard L.E., Hansen W., Linhjell D., Painter T.J. (2004). Aging of Oil-Impregnated Paper in Power Transformers. IEEE Trans. Power Deliv..

[5] Zhang E., Liu J., Zhang C., Zheng P., Nakanishi Y., Wu T. (2023). State-of-Art Review on Chemical Indicators for Monitoring the Aging Status of Oil-Immersed Transformer Paper Insulation. Energies.

[6] Mharakurwa E.T. (2022). In-Service Power Transformer Life Time Prospects: Review and Prospects. J. Electr. Comput. Eng..

[7] Tian Y., Li Z., Wang S., Zhu G., Shi H., Wang Y., Niu B., Zhu Y., Huang X. (2024). Research on Infrared Spectroscopy Detection of Furfural Content in Transformer Oil Based on Acetonitrile Extraction. Meas. Sci. Technol..

[8] Manuel G.-M.J., Cecilia G.-G., Elías B.-B.J., Gabriela M.-C., Elizabeth L.-B., Ramiro V.-R. (2019). Occurrence of Emerging Contaminants in Environmental Surface Waters and Their Analytical Methodology—A Review. Water Supply.

[9] Lin M., Lin K., Lin A., Gras R., Luong J. (2016). Ultra-trace Analysis of Furanic Compounds in Transformer/Rectifier Oils with Water Extraction and High-performance Liquid Chromatography. J. Sep. Sci..

[10] Guterres B.K., Pereira S.T.T., Oliveira R., Moonen C.G.J., Heinemann M.B., Araújo F., Palaci M., de Souza G.O., Guimarães N.S., Guimarães A.M.S. (2026). Discrimination between Mycobacterium tuberculosis and Mycobacterium bovis using Fourier transform infrared spectroscopy. One Health.

[11] Wu R., He Y., Jiang J., Wang X., Fan L. (2023). Temperature and Pressure Characteristics of a Photothermal Interference Detection System for Dissolved Acetylene in Transformer Oil. Electr. Power Eng. Technol..

[12] Zhang S., Qi Y., Tan S.P.H., Bi R., Olivo M. (2023). Molecular Fingerprint Detection Using Raman and Infrared Spectroscopy Technologies for Cancer Detection: A Progress Review. Biosensors.

[13] Wang P., Chen W., Wang J., Tang J., Shi Y., Wan F. (2020). Multigas Analysis by Cavity-Enhanced Raman Spectroscopy for Power Transformer Diagnosis. Anal. Chem..

[14] Zhang W.Q., Liu J.F., Jiang J., Zhao X.F., Fan L.D. (2024). Prediction of concentration for dissolved gas in oil based on CEEMDAN and TCN. Electr. Power Eng. Technol..

[15] Zhang Y., Zhang L., Wang L., Shao S., Tao B., Hu C., Chen Y., Shen Y., Zhang X., Pan S. (2025). Subcutaneous Depth-Selective Spectral Imaging with mμSORS Enables Noninvasive Glucose Monitoring. Nat. Metab..

[16] Kubackova J., Fabriciova G., Miskovsky P., Jancura D., Sanchez-Cortes S. (2015). Sensitive Surface-Enhanced Raman Spectroscopy (SERS) Detection of Organochlorine Pesticides by Alkyl Dithiol-Functionalized Metal Nanoparticles-Induced Plasmonic Hot Spots. Anal. Chem..

[17] Gu C., Chen W., Du L., Wang P., Wan F., Zou J. (2017). In-Situ Detection of Dissolved Furfural in Transformer Oil Based on Silver Nanosheet Surface-Enhanced Raman Spectroscopy. Proc. CSEE.

[18] Song R., Chen W., Wang Z., Zhang Z., Huang Y., Wang J. (2023). Centrifugal Extraction-Assisted Fiber-Enhanced Raman Spectroscopy for Online Detection of Trace Furfural in Oil Power Equipment. Anal. Chem..

[19] Wan F., Lei Y., Wang M., Li S., Liu R., Xia Y., Wang C., Liu H., Chen W., Wan F. (2025). Sensitive and Rapid Extraction-Free Detection of Furfural in Mineral and Natural Ester Oils by 4-ATP Modified SERS Substrate. IEEE Trans. Instrum. Meas..

[20] Jie X. (2026). SERS-Based Quantitative Detection of Furfural in Transformer Oil Using an Improved Residual Network and Transfer Learning. Spectrochim. Acta Part A Mol. Biomol. Spectrosc..

[21] Ranasinghe J.C., Sanders S.K., Wang Z., Mehrani J., Wu W., Dimitrov E., Wang X., Minns A.M., Rossi R.M., Lindner S.E. (2026). Noise Management of Surface-Enhanced Raman Spectroscopy Using Two-Dimensional Materials. ACS Sens..

[22] Wan F., Li S., Lei Y., Wang M., Liu R., Hu K., Xia Y., Chen W. (2024). Two-Step Machine Learning-Assisted Label-Free Surface-Enhanced Raman Spectroscopy for Reliable Prediction of Dissolved Furfural in Transformer Oil. Spectrochim. Acta Part A Mol. Biomol. Spectrosc..

[23] Shi H., Chen W., Wan F., Du L., Zhang S., Zhou W., Zhang J., Huang Y., Zhu C. (2018). Application of Self-Assembled Raman Spectrum-Enhanced Substrate in Detection of Dissolved Furfural in Insulating Oil. Nanomaterials.

[24] Pei Z., Ji C., Shao M., Wu Y., Zhao X., Man B., Li Z., Yu J., Zhang C. (2025). Enrichment Strategies in Surface-Enhanced Raman Scattering: Theoretical Insights and Optical Design for Enhanced Light-Matter Interaction. Opto-Electron. Sci..

[25] Yan C. (2025). A Review on Spectral Data Preprocessing Techniques for Machine Learning and Quantitative Analysis. iScience.

[26] Wang W., Xia L., Xiao X., Li G. (2024). Recent Progress on Microfluidics Integrated with Fiber-Optic Sensors for On-Site Detection. Sensors.

